# Case Report: Skeletal and cardiovascular alterations compatible with a connective tissue disorder in an elderly dog

**DOI:** 10.3389/fvets.2025.1662596

**Published:** 2025-11-28

**Authors:** Bengü Bilgiç, Lora Koenhemsi, Michela Pugliese, Abdullah Altındal, Mehmet Erman Or

**Affiliations:** 1 Department of Internal Medicine, Faculty of Veterinary Medicine, Istanbul University-Cerrahpasa, Istanbul, Türkiye; 2 Department of Veterinary Sciences, Faculty of Veterinary Medicine, University of Messina, Messina, Italy

**Keywords:** aortic dilation, aortic regurgitation, deformities, syndrome, dog

## Abstract

A 15-year-old intact female Siberian Husky was referred for hindlimb paresis, anorexia, and cachexia. Cardiac auscultation revealed a grade 5/6 systolic murmur, with maximum intensity over the right hemithorax. Right lateral thoracic radiography revealed an undulating thoracic aorta with a normal vertebral heart scale. The patient had a left-sided scoliosis deformity at the lower thoracic and upper lumbar levels. In addition, sternum deformity revealed pectus excavatum, with no history of trauma in the anamnesis. Echocardiographic examination revealed aortic regurgitation (5.29 m/sn) and dilation of the aortic root and proximal ascending aorta in the parasternal long-axis view. The left ventricle was mildly enlarged, and the left atrial diameter was within reference ranges. This case describes an uncommon combination of skeletal and cardiovascular abnormalities in an elderly dog, raising the suspicion of a connective tissue disorder. However, in the absence of molecular or histopathological confirmation, the diagnosis remains presumptive. The case highlights the need for further genetic investigations and the establishment of specific diagnostic criteria for connective tissue fragility syndromes in veterinary medicine.

## Introduction

Connective tissue integrity plays a critical role in maintaining both skeletal and cardiovascular structure and function. In geriatric dogs, age-related weakening of connective tissues may clinically manifest as progressive loss of skeletal muscle mass and strength, a condition known as senile sarcopenia, which can lead to reduced mobility and generalized weakness (1).

Similar degenerative alterations may also occur in large elastic arteries such as the aorta, where cumulative mechanical stress and disruption of the extracellular matrix can promote vascular remodeling and dilation (2). These alterations may predispose the aorta to dilation and aneurysm formation. Although aortic dilations and aneurysms are rare in dogs, they are clinically significant due to the risk of rupture or dissection (3). Reported causes include systemic hypertension, congenital abnormalities such as bicuspid aortic valve, infectious or inflammatory aortitis, and spontaneous aortic dissection (4–8). In older animals, histopathological changes such as intimal thickening, medial fibrosis, fragmentation of elastic fibers, smooth muscle cell loss, and microcalcification can further weaken the aortic wall and predispose it to aneurysmal dilation (9, 10).

Another disorder that can lead to concurrent skeletal and aortic abnormalities is Marfan syndrome (MFS), a heritable connective tissue disease caused by mutations in the fibrillin-1 (FBN1) gene, which plays a key role in microfibril and elastic fiber formation (11). In human medicine, diagnosis is based on the Ghent nosology, in which aortic root dilation or dissection and ectopia lentis constitute major diagnostic criteria, complemented by systemic findings involving the skeleton, skin, lungs, and other organ systems (12, 13).

While the clinical and genetic characteristics of MFS and MFS-like phenotypes are well defined in humans, the veterinary literature remains extremely limited, and the spectrum of connective tissue disorders in dogs is still poorly characterized. The present case report describes the concurrent presence of skeletal and cardiovascular abnormalities consistent with connective tissue weakness in an elderly dog, emphasizing the need for increased clinical awareness and further characterization of such conditions in veterinary medicine.

## Case report

A 15-year-old intact female Siberian Husky was referred to our faculty clinic. Written informed consent was obtained from the owner for the participation of their animal in this case report.

Previously, the dog had experienced chronic, episodic hindlimb weakness for six months, accompanied by anorexia, and was diagnosed mitral and aortic valve regurgitation, as well as intra-abdominal and extra-abdominal masses. Accordingly, prednisolone at a dose of 2 mg/kg q12h and enalapril at a dose of 0.25 mg/kg q12h were prescribed by another clinic after the first examination, and no clinical response was observed.

During a recheck examination at our clinic, the patient had a very low body condition score (2/9) and weighed 11.6 kg. The dog was not able to use any of the extremities. The rectal temperature was 38.6 °C. Cardiac auscultation revealed a grade 5/6 systolic murmur, with maximum intensity over the right hemithorax. Heart rate was 80 beats/min, and respiration rate was 23 breaths/min. Femoral pulse rhythm, frequency, and quality were normal on palpation. Blood pressure measured in the right forelimb using a PetTrust^®^ blood pressure monitor was 152 mmHg (systolic), 97 mmHg (diastolic), and 115 mmHg (mean arterial pressure).

A complete blood count was performed using the Fujifilm^®^ VH5R, and a biochemistry panel was analyzed using the Fujifilm^®^ DRI-CHEM NX600 device. Leukocytosis was detected at 22.97 10 (9)/L (reference range 6.00–17.00 10^9^/L), with neutrophils increased to 19.479 10^9^/L (reference range 3.62–11.32 10^9^/L). Normocytic, hyperchromic anemia was observed with decreased red blood cells at 3.74 10^12^/L (5.10–8.50 10^12^/L) and hematocrit at 25.5% (reference range 36.00–56.00). The biochemistry and electrolyte panel were within reference ranges, except for mild hypoalbuminemia at 2.5 g/dL (reference range 2.60–4.00), hypocalcemia at 9 mg/dL (reference range 9.30–12.10), and an increased lipase concentration of 375 U/L (reference range 10.00–160.00).

**Figure 1:**
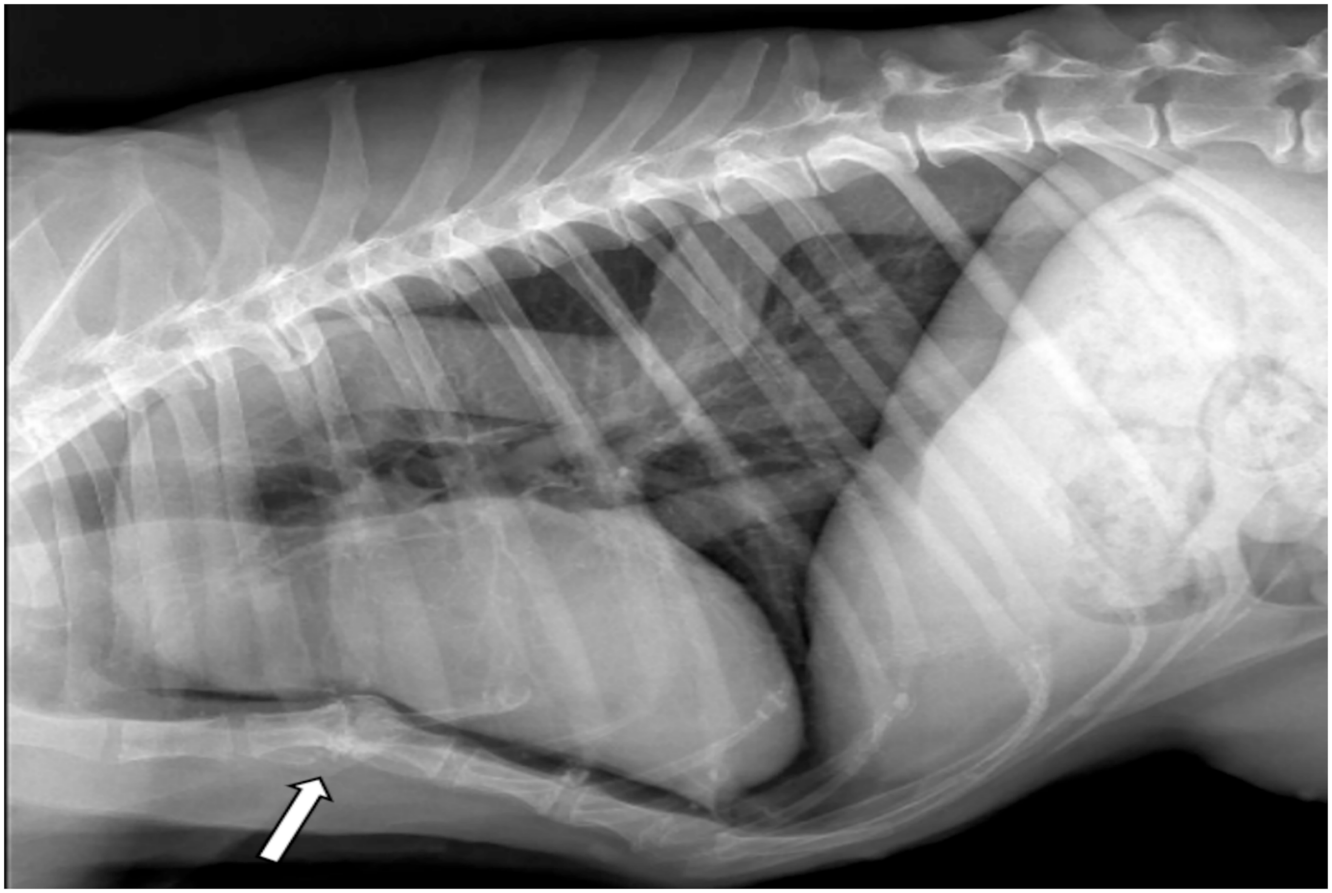
Right lateral thoracic radiograph showing dilation of the proximal aspect of the aortic arch and the descending aorta extending toward the thoracic vertebrae. Note the ventrodorsal deviation of the sternum (arrow).

Right lateral thoracic radiography revealed an undulating thoracic aorta. Dilation was observed at the level of the aortic root and ascending aorta. However, the vertebral heart score (VHS) was 9 (reference range 8.5–10.6) (13). The patient had a left-sided scoliosis deformity at the lower thoracic and upper lumbar levels. In addition, sternum deformity revealed pectus excavatum (Figure 1). There was no history of trauma in the anamnesis.

**Figure 2:**
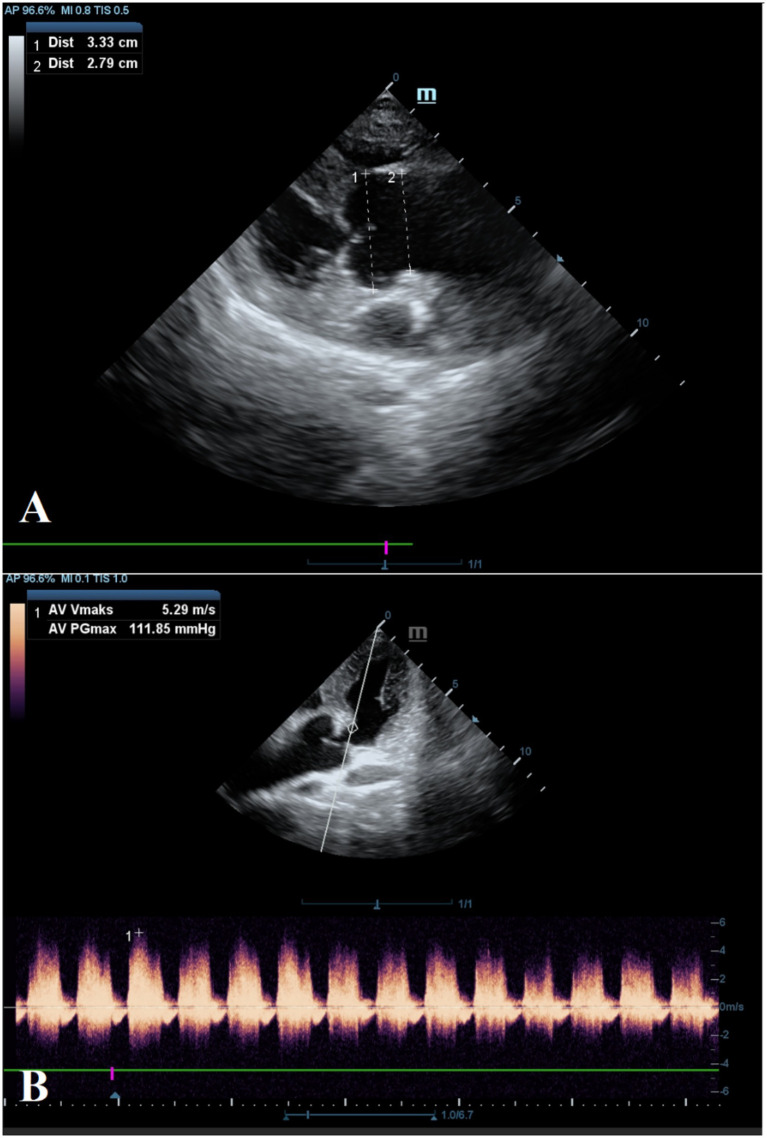
Echocardiographic evaluation of the aorta. Dimensions of the sinuses of Valsalva (1) and the sinotubular junction (2) **(A)**. Aortic valvular regurgitation with a maximal velocity of 5.29 m/s and a pressure gradient of 111.85 mmHg **(B)**.

Electrocardiographic evaluation revealed a prolonged PR interval of 0.28 s. Echocardiographic examination revealed aortic regurgitation (5.29 m/sn), a maximal pressure gradient of 111.85 mmHg, and dilation of the aortic root and proximal ascending aorta in the parasternal long-axis view (Figure 2). The left ventricle was mildly enlarged, and the left atrial diameter was within reference ranges (Table 1). The aortic valves, commissures, and rings reflected natural anatomical structures. Mitral valve degeneration with mild regurgitation (<2 m/sn) and tricuspid valve regurgitation (2.73 m/s) was observed, without right atrial enlargement. No cardiac defect was detected.

**Table 1:** Echocardiographic findings with the corresponding reference values (22, 23).

	Results	Reference value
M-mode measurement		
IVSd (cm)	0.79	0.52–1.05
LVIDd (cm)	4.46	2.64–3.84
LVPWd (cm)	0.75	0.52–1.07
IVSs (cm)	1.46	0.78–1.43
LVIDs (cm)	2.25	1.55–2.76
LVPWs (cm)	1.31	0.83–1.51
EF (%)	81.09	40–100
FS (%)	49.58	33.70–45.90
Doppler measurement		
MV E Vel (cm/sn)	57.71	≤100
MV A Vel (cm/sn)	65.54	≤75
TV E Vel (cm/sn)	171.81	≤80
TV A Vel (cm/sn)	73.33	≤60
AV Vmax (m/sn)	5.29	≤1.7
PV Vmax (m/sn)	0.73	≤1.3
Two-dimensional measurement		
Ao (cm)	3.24	1.47–2.24
LA (cm)	2.32	1.39–2.29
LA/Ao	0.71	0.83–1.13

IVSd, interventricular septum thickness at end-diastole; LVIDd, left ventricular internal dimension at end diastole; LVPWd, left ventricular posterior wall thickness at end-diastole; IVSs interventricular septum at end-systole thickness at end-systole; LVIDs, left ventricular internal dimension at end-systole; LVPWs, left ventricular posterior wall thickness at end-systole; EF, Ejection fraction; FS, fractional shortening; MV E Vel, mitral valve E velocity; MV A Vel, mitral valve A velocity; TV E Vel, tricuspid valve E velocity; TV A Vel, tricuspid valve A velocity; AV Vmax, aortic valve maximum velocity; PV Vmax, pulmoner valve maximum velocity; Ao, Aorta; LA, left atrium diameter; LA/Ao, ratio of the left atrial to the aortic annulus.

**Figure 3:**
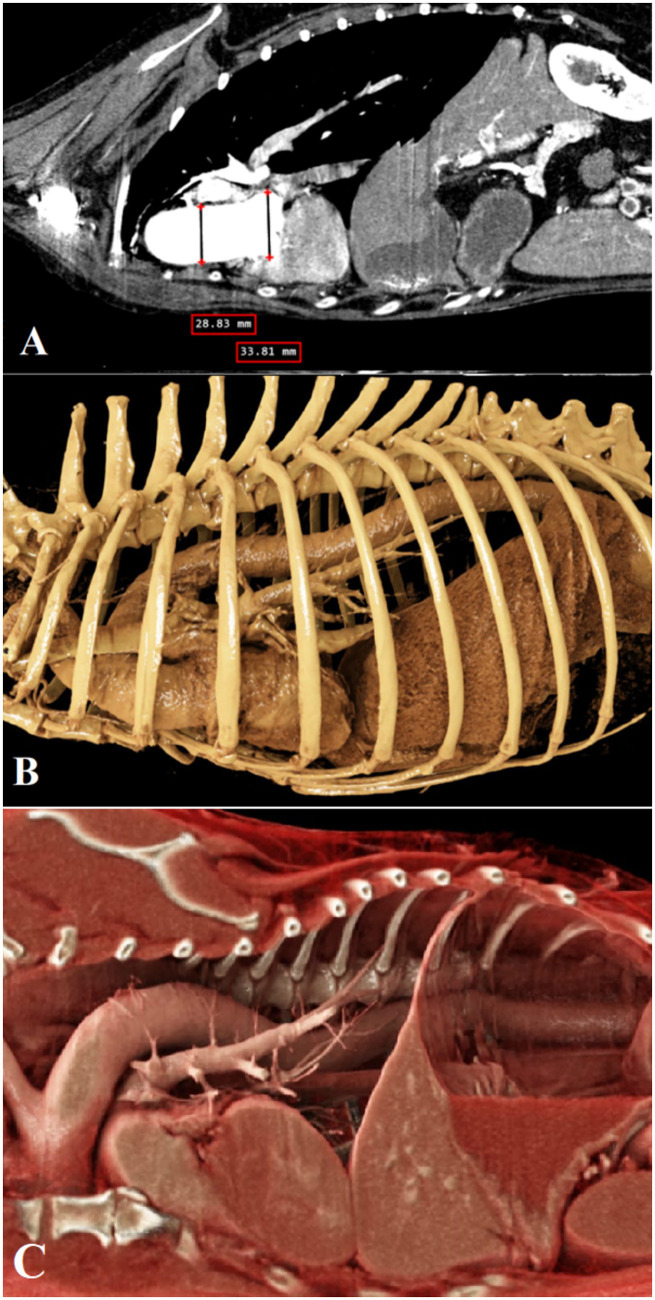
Sagittal views of the aorta on CT images: contrast-enhanced **(A)** and cinematic rendering **(B,C)**. The diameter at the level of the sinuses of Valsalva was 33.81 mm, and the ascending aorta at the tubular segment was 28.83 mm.

Contrast CT scanning showed concentric thickening of the myocardial wall. The diameter at the level of the sinuses of Valsalva was 33.3 mm, at the tubular segment of the ascending aorta was 27.9 mm, at the aortic arch was 25 mm, and at the level of the descending aorta was 18 mm. In addition, no signs of dissection were observed in the aorta or its branches on CT evaluation (Figure 3).

The dog was euthanized due to severe pain and a poor prognosis associated with multiple abdominal masses. Necropsy was not performed at the owner’s request.

## Discussion

This case describes an uncommon combination of skeletal and cardiovascular abnormalities in an elderly dog, in which degenerative aortic changes and associated regurgitation were the predominant cardiac abnormalities.

Among the most common acquired etiologies of aortic regurgitation are degenerative or atherosclerotic changes of the aortic valve associated with aging, systemic hypertension, and hyperlipidemia. These processes impair cusp coaptation during diastole, resulting in retrograde flow from the aorta into the left ventricle (14). Age-related factors, in particular, contribute to reduced leaflet elasticity, increased leaflet thickness due to the accumulation of adipose tissue between the fibrosa and ventricularis layers, deposition of extracellular matrix components and cellular degradation products, and increased collagen fiber density. Collectively, these changes lead to leaflet stiffening and reduced compliance. Over time, this degenerative progression results in valve thickening, rigidity, and calcification, ultimately causing progressive narrowing of the valvular orifice (15). Chronic exposure to high-velocity, turbulent blood flow distal to the valve promotes further vascular remodeling, especially at the level of the sinuses of Valsalva and the sinotubular junction (16). The severe aortic regurgitation and distal aortic dilation observed in the present case are consistent with these pathological mechanisms. However, due to the owner’s refusal of necropsy, histopathological examination of the aortic wall could not be performed.

Aortic remodeling associated with loss of aortic elasticity and aortic root dilation secondary to systemic hypertension has been previously reported in dogs (7, 17). In the current case, despite chronic aortic regurgitation with a regurgitant jet velocity of approximately 5.29 m/s, the patient remained normotensive. In human medicine, aortic regurgitation in normotensive individuals has been associated with advanced age, female sex, fibro-calcific changes of the aortic valve, and low body mass index (18). In this case, despite the presence of both aortic and mitral regurgitation, systemic arterial blood pressure and echocardiographic indicators of left heart filling pressures remained within normal limits. This suggests that the patient was still in a compensated phase, during which forward stroke volume is preserved and both the left ventricle and left atrium exhibit increased compliance.

Another important differential diagnosis capable of producing similar pathological changes is MFS. The most common cardiovascular manifestation of MFS in humans is progressive dilation of the aortic root originating at the sinuses of Valsalva, followed by aneurysm formation and aortic dissection (18, 19). In this case, the greatest aortic enlargement was similarly located at the sinuses of Valsalva. Gradual, asymptomatic expansion of the aortic root can weaken the arterial wall and predispose it to acute ascending aortic dissection; however, no evidence of dissection was observed here. Additional systemic manifestations commonly reported in human MFS include excessive elongation of tubular bones, disproportionate limb length, chest wall deformities such as pectus excavatum or pectus carinatum, connective tissue laxity, joint hypermobility, and progressive scoliosis (12). In this dog, left-sided scoliosis at the mid-thoracic and cranial lumbar vertebrae was confirmed by CT, and pectus excavatum with additional spinal deformities was documented. Aortic undulation and dorsal deviation of the descending aorta may reflect elastic fiber fragmentation and medial degeneration, features frequently reported in MFS.

The Ghent scoring system used for MFS diagnosis in humans has not been adapted or validated for veterinary patients. In the present case, the cumulative systemic score was 6, consisting of pectus excavatum (2), hindlimb deformities (2), scoliosis (1), and mitral valve prolapse (1). However, as this scoring system is human-specific, its applicability to dogs remains uncertain (12). In addition, genetic confirmation via FBN1 testing, a key diagnostic tool in MFS, is not widely available in veterinary medicine; therefore, diagnosis in this case relied on clinical and imaging findings.

Considering the dog’s advanced age and low body condition score, some of the observed gait abnormalities were likely attributable to age-related muscle wasting and connective tissue disorder. Similarly, skeletal deviations such as limb angulation and scoliosis may represent degenerative changes associated with aging. While the findings raise suspicion of MFS, the diagnosis remains presumptive. No validated diagnostic algorithm currently exists for MFS-like disorders in dogs, and genotype–phenotype associations remain insufficiently defined.

Aortic dissections are rarely reported in dogs but are more frequently described in humans. Clinical signs in affected dogs may include paraparesis, arterial thromboembolism, hypertension, collapse, seizures, forelimb lameness, respiratory distress, and opisthotonus. Radiographically, a dilated or irregular aortic contour or focal bulging of the ascending aorta may suggest dissection. In the present case, despite hindlimb weakness as the primary complaint, neither echocardiography nor CT revealed evidence of dissection. A previously reported case of MFS-associated aortic dissection in a Great Dane demonstrated similar findings, including mitral and tricuspid regurgitation, severe aortic insufficiency, and ascending aortic dilation (20). Furthermore, a case of annuloaortic ectasia in a young Newfoundland dog demonstrated persistent aortic dilation without dissection or hemodynamic collapse, and immunofluorescence analysis revealed reduced fibrillin-1 deposition in the aortic wall, supporting a role for connective tissue fragility in canine aortic enlargement (21). These comparative findings suggest that the aortic dilation observed in the present case may reflect underlying structural weakness of connective tissue.

Bicuspid aortic valve was considered a differential diagnosis; however, echocardiographic assessment demonstrated normal cusp and commissural anatomy. In addition, this condition is typically observed in younger animals, making it unlikely in this case.

A major limitation of this case report is the absence of necropsy, histopathology, and genetic testing (FBN1), which prevents a definitive diagnosis and necessitates reliance solely on clinical and imaging findings.

## Conclusion

This case report describes a rare clinical presentation characterized by concurrent skeletal deformities, aortic root dilation, and advanced valvular insufficiencies in an elderly dog. Although the findings raise suspicion of an underlying connective tissue fragility disorder, age-related degenerative changes remain an important differential consideration. The lack of standardized diagnostic criteria and accessible genetic testing for Marfan-like phenotypes in veterinary medicine poses a significant challenge to establishing a definitive diagnosis. Therefore, in dogs presenting with similar clinical and imaging features, connective tissue disorders should be considered in the differential diagnosis, and, when feasible, further histopathological and molecular analyses are recommended to support diagnostic confirmation.

## Data Availability

The raw data supporting the conclusions of this article will be made available by the authors, without undue reservation.

## References

[1] Hutchinson D Sutherland-Smith J Watson AL Freeman LM. Assessment of methods of evaluating sarcopenia in old dogs. Am J Vet Res. (2012) 73:1794–800. doi: 10.2460/ajvr.73.11.1794, PMID: 23106466

[2] Redheuil A Yu WC Wu CO Mousseaux E De Cesare A Yan R . Reduced ascending aortic strain and distensibility: earliest manifestations of vascular aging in humans. Hypertension. (2010) 55:319–26. doi: 10.1161/HYPERTENSIONAHA.109.141275 20065154 PMC3035625

[3] Chetboul V Tessier D Borenstein N Delisle F Zilberstein L Payen G . Familial aortic aneurysm in Leonberg dogs. J Am Vet Med Assoc. (2003) 223:1159–62. doi: 10.2460/javma.2003.223.1159, PMID: 14584747

[4] Waldrop JE Stoneham AE Tidwell AS Jakowski RM Rozanski EA Rush JE. Aortic dissection associated with aortic aneurysms and posterior paresis in a dog. J Vet Intern Med. (2003) 17:223–9. doi: 10.1111/j.1939-1676.2003.tb02438.x, PMID: 12683625

[5] Fernandes S Khairy P Graham DA Colan SD Galvin TC Sanders SP . Bicuspid aortic valve and associated aortic dilation in the young. Heart. (2012) 98:1014–9. doi: 10.1136/heartjnl-2012-301773, PMID: 22668868

[6] Katabathina VS Restrepo CS. Infectious and noninfectious aortitis: cross-sectional imaging findings. Semin. Ultrasound CT MRI. (2012). 33: 207–21. doi: 10.1053/j.sult.2011.12.001 22624966

[7] Sangwan T Saini N Anand A Bisla A. Thoracic and abdominal aortic alterations in dogs affected with systemic hypertension. Res Vet Sci. (2023) 159:133–45. doi: 10.1016/j.rvsc.2023.04.017, PMID: 37141684

[8] Milewicz DM Dietz HC Miller DC. Treatment of aortic disease in patients with Marfan syndrome. Circulation. (2005) 111:150–7. doi: 10.1161/01.CIR.0000155243.70456.F4 15781745

[9] Yang C Kohnken R. Age-related changes in the canine aorta. Vet Pathol. (2020) 58:376–83. doi: 10.1177/0300985820970499, PMID: 33205711

[10] Seeburun S Wu S Hemani D Pham L Ju D Xie Y . Insights into elastic fiber fragmentation: mechanisms and treatment of aortic aneurysm in Marfan syndrome. Vasc Pharmacol. (2023) 153:107215. doi: 10.1016/j.vph.2023.107215, PMID: 37640090 PMC10872825

[11] Coelho SG Almeida AG. Marfan syndrome revisited: from genetics to clinical practice. Rev Port Cardiol. (2020) 39:215–26. doi: 10.1016/j.repc.2019.09.008, PMID: 32439107

[12] Loeys BL Dietz HC Braverman AC Callewaert BL De Backer J Devereux RB . The revised Ghent nosology for the Marfan syndrome. J Med Genet. (2010) 47:476–85. doi: 10.1136/jmg.2009.072785, PMID: 20591885

[13] Buchanan JW Bücheler J. Vertebral scale system to measure canine heart size in radiographs. J Am Vet Med Assoc. (1995) 206:194–9. doi: 10.2460/javma.1995.206.02.194, PMID: 7751220

[14] Flint N Wunderlich NC Shmueli H Ben-Zekry S Siegel RJ Beigel R. Aortic regurgitation. Curr Cardiol Rep. (2019) 21:65. doi: 10.1007/s11886-019-1144-6, PMID: 31161305

[15] Singh R Strom JA Ondrovic L Joseph B VanAuker MD. Age-related changes in the aortic valve affect leaflet stress distributions: implications for aortic valve degeneration. J Heart Valve Dis. (2008) 17:290–8. PMID: 18592926

[16] Wilton E Jahangiri M. Post-stenotic aortic dilatation. J Cardiothorac Surg. (2006) 1:7. doi: 10.1186/1749-8090-1-7, PMID: 16722611 PMC1464384

[17] Corda A Corda F Caivano D Saderi L Sotgiu G Mollica A . Ultrasonographic assessment of abdominal aortic elasticity in hypertensive dogs. J Vet Intern Med. (2020) 34:2337–44. doi: 10.1111/jvim.15891, PMID: 32949191 PMC7694867

[18] Palmieri V Bella JN Arnett DK Roman MJ Oberman A Kitzman DW . Aortic root dilatation at sinuses of valsalva and aortic regurgitation in hypertensive and normotensive subjects: the hypertension genetic epidemiology network study. Hypertension. (2001) 37:1229–35. doi: 10.1161/01.HYP.37.5.1229, PMID: 11358933

[19] Milewicz DM Braverman AC De Backer J Morris SA Boileau C Maumenee IH . Marfan syndrome. Nat Rev Dis Primers. (2021) 7:64. doi: 10.1038/s41572-021-00298-7, PMID: 34475413 PMC9261969

[20] Lenz JA Bach JF Bell CM Stepien RL. Aortic tear and dissection related to connective tissues abnormalities resembling Marfan syndrome in a great Dane. J Vet Cardiol. (2015) 17:134–41. doi: 10.1016/j.jvc.2015.01.001, PMID: 25890485

[21] Côté E Zhang RM Kaiser N Reinhardt DP Martin CK. Annuloaortic ectasia in a dog: long-term follow-up and immunofluorescent study. Vet Q. (2021) 41:280–91. doi: 10.1080/01652176.2021.1961039, PMID: 34607531 PMC8526017

[22] Boon JA. Appendix two canine In: Boon JA, editor. Veterinary echocardiography. USA: John Wiley & Sons Inc. (2011). 678–704.

[23] Palacio MFD Bernal LJ Bayon A Bernabe A Oca RMD Seva J. Arrhythmogenic right ventricular dysplasia/cardiomyopathy in a Siberian husky. Small Anim Pract. (2001) 42:137–42. doi: 10.1111/j.1748-5827.2001.tb02010.x, PMID: 11303856

