# Correction: Case Report: Skeletal and cardiovascular alterations compatible with a connective tissue disorder in an elderly dog

**DOI:** 10.3389/fvets.2025.1768165

**Published:** 2026-01-12

**Authors:** Bengü Bilgiç, Lora Koenhemsi, Michela Pugliese, Abdullah Altindal, Mehmet Erman Or

**Affiliations:** 1 Department of Internal Medicine, Faculty of Veterinary Medicine, Istanbul University-Cerrahpasa, Istanbul, Türkiye; 2 Department of Veterinary Sciences, Faculty of Veterinary Medicine, University of Messina, Messina, Italy

**Keywords:** aortic dilation, aortic regurgitation, deformities, syndrome, dog

In the published article, an incorrect **Funding** statement was provided. The correct funding statement reads:

The author(s) declared that financial support was received for this work and/or its publication. The authors acknowledge support from the University of Messina through the APC initiative.

The original version of this article has been updated.

